# Technical considerations for positioning and placement of a transperineal ultrasound probe during prostate radiotherapy

**DOI:** 10.1002/jmrs.439

**Published:** 2020-10-05

**Authors:** Eric Pei Ping Pang, Kellie Knight, Ronnie Wing‐Kin Leung, Michael Lian Chek Wang, Jason Wei Siang Chan, Gee Keng Low, Irene Kai Ling Seah, Muhammad Asri Bin Atan, Jairia Yih Huei Chai, Grace Chuk‐Kwan Ng, Tommy Chung‐Sing Yang, Jeffrey Kit Loong Tuan

**Affiliations:** ^1^ Division of Radiation Oncology National Cancer Centre Singapore Singapore Singapore; ^2^ Faculty of Medicine, Nursing and Health Sciences Department of Medical Imaging & Radiation Sciences Monash University Clayton VIC Australia; ^3^ Department of Clinical Oncology Tuen Mun Hospital Tuen Mun, New Territories Hong Kong; ^4^ Duke‐NUS Graduate Medical School Singapore Singapore

**Keywords:** Intra‐fraction motion, Prostate radiotherapy, Real‐time, TPUS, Patient positioning

## Abstract

This technical evaluation aims to provide practice ‘how to’ guidelines for radiation therapists (RTs) when positioning a transperineal ultrasound (TPUS) probe during prostate radiotherapy. Recommendations and practical tips will be provided for the best practice in TPUS‐guided workflow to obtain optimal ultrasound images for accurate interpretation and registration of the prostate gland. This will assist the RTs in making consistent and accurate clinical decision in an ultrasound‐guided radiotherapy workflow for prostate treatment. The implementation process and the associated successes and challenges will also be described to assist institutions who may be investigating the potential of implementing this system.

## Graphical abstract

This technical note aims to provide practice guidelines for radiation therapists (RTs) when positioning a transperineal ultrasound (TPUS) probe during prostate radiotherapy. Recommendations and practical tips were provided for best practice in TPUS‐guided workflow to obtain optimal ultrasound images for accurate interpretation and registration of the prostate gland. This will assist the RTs in making consistent and accurate clinical decision in an ultrasound‐guided radiotherapy workflow for prostate treatment.

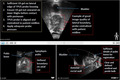

## Purpose

Clinical application of the four‐dimensional (4D) TPUS Clarity^®^ system (Elekta AB Stockholm, Sweden) as a form of non‐invasive imaging modality for localising the prostate gland and monitoring its motion during radiotherapy have been widely explored and validated.^1^, ^2^, ^3^, ^4^, ^5^, ^6^, ^7^, ^8^ The importance of probe placement for optimal transperineal ultrasound (TPUS) images for the purpose of image‐guided radiotherapy (IGRT) workflow in prostate radiotherapy was previously emphasised by Grimwood et al.^1^ We aim to provide recommendations and technical guidelines for the placement of TPUS probe for patients undergoing prostate radiotherapy. The associated implementation process, achievements and challenges will also be described. Clinical examples will be used to illustrate potential issues that can arise due to sub‐optimal placement of the autoscan TPUS probe.

## Significance of optimal TPUS image quality

The expected superior image quality using a TPUS technique compared to trans‐abdominal ultrasound (TAUS) can be attributed to the shorter scan path length. ^9^ The design of an autoscan ultrasound probe is capable of performing a mechanical sweep within the probe housing while mounted onto the autoscan probe kit (ASPK) baseplate which enables tracking of the intra‐fraction prostate motion during radiotherapy. ^1^, ^10^ The clinical value of an optimal TPUS image can be appreciated in its application during CT simulation, treatment planning and daily IGRT.

## TPUS clinical protocol background

At CT simulation, patients are positioned with the autoscan TPUS probe positioned against the perineum to acquire the reference TPUS image for image registration. Obtaining a good quality reference TPUS image is paramount as this step remains the source of reference and for motion‐tracking accuracy for subsequent radiotherapy processes. Once the reference TPUS image is acquired during the same CT simulation, the image data are then exported together with the CT images to the automatic fusion and contouring (AFC) workstation for localisation of the TPUS prostate before being exported to the treatment planning system. The registered CT/TPUS images, even in the absence of MRI fusion, can be used to improve the visualisation of the prostate gland especially in the apex of the prostate.^11^

During daily IGRT at the treatment unit, TPUS acts as a pre‐treatment set up verification tool and provides continuous real‐time intra‐fraction prostate monitoring capability. TPUS is a non‐invasive IGRT platform for the tracking of the prostate (especially in the case of hypo‐fractionated SBRT to reduce the planning target margin),^12^ enabling automatic beam holding when gross prostate motion exceeds the pre‐determined tolerance which can be individualised at the planning stage. The following recommendations provide RTs guidelines for routine practice to achieve optimal and reproducible images when in using TPUS.

## Guidelines on the placement of TPUS probe during CT simulation and daily treatment

### CT simulation

**Figure 1:**
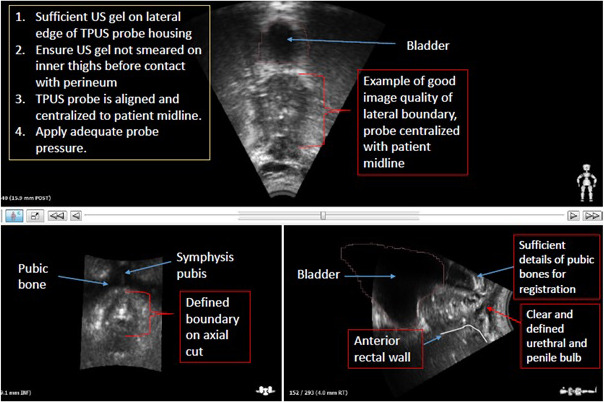
Visual illustration of the guidelines used to achieve optimal TPUS image quality.

**Figure 2:**
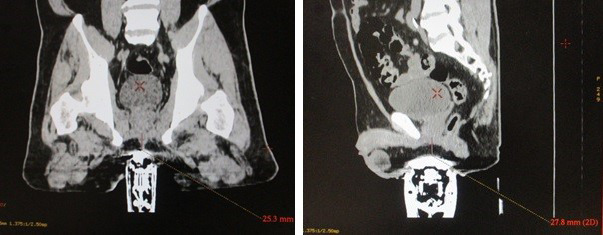
Illustration of the TPUS probe distance from the inferior boundary of the prostate gland as seen on the final planning scan.

1. Apply an adequate amount of high viscosity ultrasound (US) gel on the scanning membrane of the TPUS probe. Centralise the probe position using the midline laser when mounted onto the ASPK. Ensure that the midline laser corresponds to the longitudinal plane of the probe for daily reproducibility.
2. In the event, a patient is unable to separate his thighs wide enough for the placement of the TPUS probe, remove one knee rest on the ASPK to position the TPUS probe for optimal contact with the perineum before repositioning the knee rest. This helps avoid smearing the US gel on the inner thighs as a lack of gel may introduce an undesirable air interface, negatively impacting image quality.
3. Adjust settings on the ultrasound acquisition cart to obtain a satisfactory set of TPUS images (Fig. 1). Adjust the probe height and angle, gain, brightness and contrast to obtain the optimal TPUS image quality as summarised in Figure 1. Optimal starting position of the probe height and angle can be determined on the sagittal view by locating the scan window between the symphysis pubis and anterior rectal wall. Next, tab directly on the screen to focus on the prostate gland region. The subsequent acquisition of TPUS images during daily treatment will be based on these image settings in the TPUS system.
4. Reference TPUS images should be acquired at the CT origin with corresponding sagittal laser offset (if any) before saving the acquired reference TPUS images (practice is dependent on departmental workflow). This will ensure that both the CT/TPUS images were acquired at the same DICOM coordinates to minimise image registration errors after exporting to the AFC workstation for contouring of the TPUS reference prostate volume.
5. Perform a cone‐down volume of the CT scan to check for organ‐at‐risk (OAR) volumes (i.e. rectum and bladder). A minimum 2.5 cm probe surface to the prostate gland distance is recommended before proceeding with the final CT scan (Fig. 2). A reconstructed sagittal view is ideal for assessing this distance and to ensure sufficient physical distance for adequate dose fall off (accounting for up to 1 cm margin expansion from the prostate gland) can be obtained and safely reproduced during daily treatment positioning to avoid any undesirable skin toxicities. The probe pressure can be reduced if optimal image quality can be achieved. May be necessary to adjust the probe distance using the mechanical dial on the ASPK and verify that the quality of the TPUS image is still acceptable.
6. In the assessment process, a patient may not be a suitable candidate for TPUS‐guided workflow when
  1. image quality is sub‐optimal compared to Fig. 1.
  2. the minimum probe surface distance to the prostate gland (≥2.5 cm) cannot be achieved.
7. Ensure adequate bladder filling (>200 cm^3^) (practice is dependent on departmental workflow).^13^
8. Reduce the time lapse between the CT/TPUS images by acquiring the TPUS images immediately before or immediately after the final CT scan to avoid gross movement which may affect subsequent registration of the CT/TPUS images.
9. All the TPUS images should be acquired at CT origin so that the CT/TPUS images will be automatically fused to the same DICOM origin when imported to the AFC workstation.
10. Measure and record TPUS set up measurements and readings. This is important to ensure set up reproducibility in the event of TPUS Clarity^®^ system downtime during treatment.

### Daily treatment

**Figure 3:**
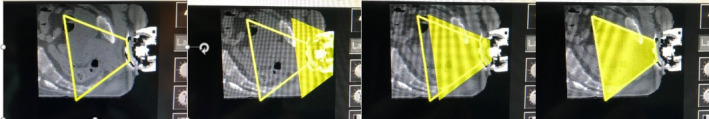
Illustration of the probe positioning guidance (yellow‐filled region) during treatment with reference position (yellow boundary) from CT simulation.

1. During daily treatment, repeat steps 1 and 2 (described under CT Simulation).
2. Align the probe angle (shaded triangle) to that of the yellow triangle at the TPUS acquisition cart. Do not push the probe beyond the yellow boundary as this may compress the perineal surface into the high dose region of the PTV (Fig. 3).
3. It is strongly recommended that image registration processes are verified by two RTs to reduce the risk of registration errors. Identify corresponding anatomical features (i.e. prostate calcification, urethra) on both the reference and treatment TPUS images prior to registration of the reference prostate gland contour on the treatment TPUS images. If the TPUS probe position is not properly reproduced during treatment, the anatomical features (i.e. prostate calcifications) may appear differently on the treatment TPUS.
4. During monitoring of prostate motion, if gross prostate displacement (>5 mm) is observed on the TPUS system during the acquisition or image registration of the CBCT, RTs should investigate the reason for the gross displacement. Repeat or abort the CBCT in the event of non‐transient displacement as this would mislead the subsequent derived CBCT shifts. From our experience, displacements could be attributed to patient coughing, urinary urgency or voiding on treatment couch.

## Implementation process and the associated success and challenges

Prior to implementation, a study, registered on the National Institutes of Health (NIH) clinical trial registry (ID: NCT02408497), was designed and conducted between 2015 and 2018 to evaluate the 4D TPUS Clarity system in the following aspects; 1) robustness of immobilisation system and favourable patients’ acceptance, 2) operator dependency and system reliability, 3) synthesis of duration‐dependent margins using data acquired on motion, 4) relationship of intra‐fraction motion and OARs and 5) minute‐by‐minute association and impact of prostate displacement on duration‐dependent margin for prostate radiotherapy.

### Five papers were published with following results

1. The clarity immobilisation system (CIS) demonstrated stability and reproducibility in prostate treatment set up comparable to the traditional leg immobiliser (LI).^14^
2. The median (range) intra‐observer variation was ≤2 mm in 93.3% (60%‐100%) of cases (maximum deviation 4.9 mm). The magnitude of observer variation appeared to be influenced by training and/or the length of user experience. 4D TPUS is a promising non‐invasive ultrasound (US)‐based IGRT solution for daily treatment set up with minimal inter‐ and intra‐observer variation.^15^
3. Larger anisotropic margins should be employed, particularly in the inferior and posterior directions, due to a greater magnitude of observed prostate displacement.^2^
4. A planned bladder volume >200 cm^3^ and daily filling between 82% and 113%, reduced intra‐fraction prostate displacement.^13^ The hydration protocol was well tolerated.
5. Derivation of minimum duration‐dependent margin to generate the planning target volume revealed that the required margin increases linearly in all directions within 15‐min duration, dependent on the duration of the chosen technique (IMRT/VMAT/3DCRT/proton).^16^

This study demonstrated the application and possibility to visualise real‐time prostate displacement via a non‐invasive imaging technique without additional radiation dose and allows users to intervene in times of gross intra‐fraction motion. From the findings, we have moved towards margin reduction (from 1 to 0.5 cm all round) while ensuring the prostate motion is tracked during treatment with enhanced guidelines on bladder preparation before treatment. Since clinical implementation in 2019, we have treated 180 prostate patients with real‐time TPUS monitoring including hypo‐fractionated cases (60 Gy in 20 fractions).

Although at present our standard practice still utilises daily pre‐treatment CBCT for the verification of the prostate +/‐ lymph nodes position and 4D TPUS for intra‐fraction monitoring, an alternate workflow was prepared to enable possible use of 4D TPUS for daily pre‐treatment localisation of the prostate without the use of CBCT imaging. For instance, the RTs would verify the prostate position using TPUS and apply the required couch shift in the event of CBCT downtime. The RTs would then proceed to acquire a two‐dimensional megavoltage (2D MV) portal image to verify the bony anatomy (L4/5 to pelvis) as a surrogate to the nodal position with a 0.5 cm  threshold. This is suggested with reference to the routine nodal margin expansion (0.5‐0.7 cm) during treatment planning. However, one of the key challenges to optimise the potential of the TPUS system is the lack of a validated interface between TPUS clarity system and Varian linear accelerators to allow automatic beam holding when gross prostate displacement is detected. For practicality, the threshold for manual intervention to beam hold is currently set at >1 cm (practice is currently under review).

## Potential pitfalls and lessons learnt

### a) Sub‐optimal image quality

**Figure 4:**
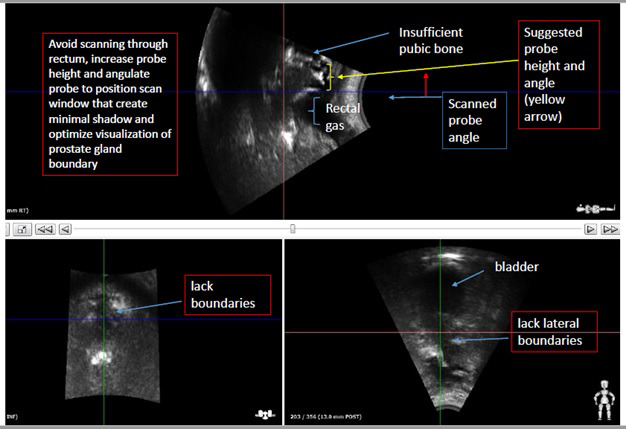
Visual example of sub‐optimal TPUS image quality.

Sub‐optimal image quality (Fig. 4) refers to the lack of distinct boundaries of the prostate gland that may compromise on the accuracy of the subsequent set up and monitoring of the prostate displacement. From our experience, the most common reason encountered for poor image quality can be attributed to poor probe contact with the perineal surface. Others include sub‐optimal selection of the scan path through inappropriate height and angle of the autoscan probe (as required in step 3 under CT simulation). Presence of rectal gas and inadequately filled bladder may also affect the visualisation of the prostate gland (Fig. 4) so patient compliance with organ filling should be confirmed.

### b) TPUS probe pressure

**Figure 5:**
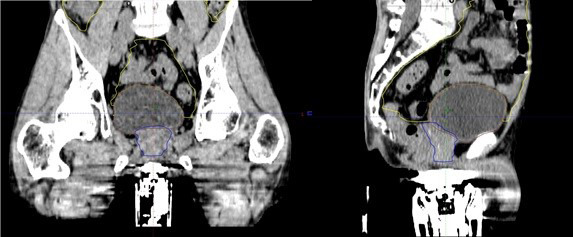
Illustration of the lack of separation between the surface of probe to the prostate gland resulting in an effective scan path from the inferior boundary of the prostate to the skin of only 1 cm.

**Figure 6:**
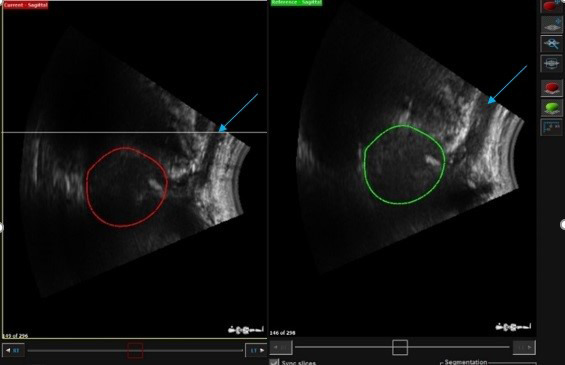
Illustration of excessive probe pressure on the penile bulb (blue arrows) (green – approved prostate gland contour from CT simulation; red – registration of approved prostate contour during treatment).

An excessively compressed perineal region due to TPUS probe pressure reduces the effective scan path from the inferior boundary of the prostate to the skin. Since traditional planning target volume (PTV) margin expansions around the prostate are 1 cm all around,^17^ with 0.6 cm posteriorly to spare the rectum, probe pressure may compress the perineal skin surface within the inferior boundaries of the PTV. An example of when excessive pressure has been applied is shown in Figure 5. If differential probe pressure is applied during daily treatment, deformation of OARs (particularly the penile bulb) and potential dosimetric deviation from the desired treatment plan may occur (Fig. 6). In order to optimise efficient execution of the clinical workflow, adequate training and user experience are imperative to reduce intra‐ and inter‐observer variations in the interpretation of the prostate gland position on the TPUS images as reported by Pang et al.^15^ If after following step 5 (under CT simulation) adequate scan path distance still cannot be achieved, the patient should be simulated and treated without the TPUS workflow. So far, this has only occurred in <5% of the patient cohort (*n* = 180) based on the authors institutional experience and usually attributed to a small patient habitus.

## Conclusion

Recommendations and tips for TPUS‐based IGRT have been outlined to assist institutions looking to achieve optimal TPUS images for prostate localisation and monitoring during treatment. Guidance has been provided for RTs to assess suitability of the TPUS set up for the patients before finalising the patient’s position and sharing lessons learnt to help RTs avoid two common pitfalls.

## Conflict of Interest

The authors do not have any conflict of interest to declare.

## Source of Financial Support/Funding Statement

This study did not receive any financial support/funding.

## Data Sharing Statement

All data generated and analysed during this study are included in this published article.
